# C4′/H4′ selective, non-uniformly sampled 4D HC(P)CH experiment for sequential assignments of ^13^C-labeled RNAs

**DOI:** 10.1007/s10858-014-9861-z

**Published:** 2014-09-10

**Authors:** Saurabh Saxena, Jan Stanek, Mirko Cevec, Janez Plavec, Wiktor Koźmiński

**Affiliations:** 1 Biological and Chemical Research Centre (CENT III), Faculty of Chemistry, University of Warsaw, Pasteura1, 02093 Warsaw, Poland; 2 Slovenian NMR Centre, National Institute of Chemistry, Hajdrihova ulica 19, 1000 Ljubljana, Slovenia; 3 EN-FIST Centre of Excellence, Dunajska cesta 156, 1000 Ljubljana, Slovenia; 4 Faculty of Chemistry and Chemical Technology, University of Ljubljana, Aškerčeva cesta 5, 1000 Ljubljana, Slovenia

**Keywords:** RNA resonance assignment, HCP, Selective pulses, Four-dimensional NMR, Non-uniform sampling

## Abstract

A through bond, C4′/H4′ selective, “out and stay” type 4D HC(P)CH experiment is introduced which provides sequential connectivity via H4′_(i)_–C4′_(i)_–C4′_(i−1)_–H4′_(i−1)_ correlations. The ^31^P dimension (used in the conventional 3D HCP experiment) is replaced with evolution of better dispersed C4′ dimension. The experiment fully utilizes ^13^C-labeling of RNA by inclusion of two C4′ evolution periods. An additional evolution of H4′ is included to further enhance peak resolution. Band selective ^13^C inversion pulses are used to achieve selectivity and prevent signal dephasing due to the of C4′–C3′ and C4′–C5′ homonuclear couplings. For reasonable resolution, non-uniform sampling is employed in all indirect dimensions. To reduce sensitivity losses, multiple quantum coherences are preserved during shared-time evolution and coherence transfer delays. In the experiment the intra-nucleotide peaks are suppressed whereas inter-nucleotide peaks are enhanced to reduce the ambiguities. The performance of the experiment is verified on a fully ^13^C, ^15^N-labeled 34-nt hairpin RNA comprising typical structure elements.

**Electronic supplementary material:**

The online version of this article (doi:10.1007/s10858-014-9861-z) contains supplementary material, which is available to authorized users.

With the advent of several new classes of non-coding RNAs (e.g. siRNA, miRNAs) research has been heavily focused on understanding the role of RNA in cellular processes during normal and diseased states (Esteller 2011), through exploring its structure–function relationship (Briones et al. 2009; Mercer et al. 2009). Over the years, in conjunction with isotope labeling techniques, several NMR approaches (Varani et al. 1996; Wijmenga and van Buuren 1998; Furtig et al. 2003; Flinders and Dieckmann 2006) proved to be highly useful in expanding our knowledge about RNA structure, its basic structural motifs, catalysis and interactions with small molecules or proteins. However, precise structural determination of even moderately sized RNAs can still be problematic. In addition to low proton density in RNAs, these biopolymers comprise only four different nucleotides. Effectively, chemical shift dispersion is inherently low, which entails severe spectral overlaps. For non-coding RNAs a frequent lack of base stacking results in even increased crowding in the NMR spectra. Moreover, similar chemical shifts are observed for many nucleotides having similar chemical environment in helical secondary structures. Recently, automated assignment approach involving no isotope labeling (Aeschbacher et al. 2013) was proposed that requires peak lists from 2D TOCSY, 2D NOESY and natural abundance ^1^H–^13^C HSQC spectra. However, difficulty may arise while assigning regions/nuclei with irregular or limited statistics. In addition, chemical shift degeneracy or low dispersion still remain a bottleneck for such approaches. This suggests that new high dimensional techniques, resembling 4D/5D methods employed for intrinsically disordered proteins (Zawadzka-Kazimierczuk et al. 2012; Stanek et al. 2013b; Bermel et al. 2013) can be explored for the assignment of poorly resolved resonances in RNAs.

The sequential resonance assignment in RNA is usually achieved using through-space NOE-type (Nikonowicz and Pardi 1993) and/or through-bond HCP (Marino et al. 1994) experiments. The efficacy of both types of experiments is severely affected due to spectral crowding and overlaps, which increases dramatically with the size of RNA. To increase the peak resolution, experiments having HCP concatenated with HCCH-TOCSY were also proposed (Marino et al. 1995; Ramachandran et al. 1996), however, their application remained limited due to significant relaxation losses during TOCSY mixing time and limited resolution owing to relatively short maximum evolution times. In account of this, high resolution 4D C_(aro)_,C_(ribo)_-NOESY experiment (Stanek et al. 2013a) was recently reported which aimed at providing the intra and inter-nucleotide (sequential) NOE correlations in RNA. As NOE interactions are largely dependent on conformations, ambiguities and gaps may arise during the assignment of NOESY spectrum, preventing the possible correlation of genuine peaks that may be present in the spectrum. These ambiguities can largely be resolved if the spectral analysis is complemented by some through-bond experiments. For example, the 3D HCP experiment, which provides sequential connectivity via intervening ^31^P nuclei, i.e. correlating H4′_(i)_–C4′_(i)_–P_(i)_ with P_(i)_–C4′_(i−1)_–H4′_(i−1)_, has been successful in many applications. However, it suffers from severe spectral overlaps (see Fig. S1a–d in Supplementary Materials) and relies on quite poor resolution of ^31^P dimension. In principle the peaks can be resolved in ^31^P dimension but in practice it is limited by its low chemical shift dispersion (~1.8 ppm) (see Fig. S1e), which makes unambiguous assignment of peaks a challenging task even for moderately sized RNAs. Additionally, in this experiment it is very difficult to unambiguously assign intra- and inter-nucleotide peaks (see Fig. S1b, c), especially when it relates to the most crowded, H4′C4′, region among sugar carbons. The possibility of sequential correlation through other sugar carbons, i.e. C3′_(i−1)_ and C5′_(i)_ is also hampered due to weak ^31^P–C3′/5′ couplings making such peaks either absent or too weak; the problem is further augmented by peak overlaps and presence of H5′/H5″ doublets. Clearly, more advanced approaches are needed to achieve an unambiguous sequential assignment in RNAs.

To address these issues we have developed a C4′/H4′ selective, four-dimensional HC(P)CH experiment with “out and stay” type transfer. The experiment includes chemical shifts evolution of ^1^H4′s and ^13^C4′s of the adjoining nucleotides thereby linking them in a single experiment with higher peak resolution. The experiment provides sequential connectivities via H4′_(i)_–C4′_(i)_–C4′_(i−1)_–H4′_(i−1)_ correlation. The ^31^P dimension (e.g. used in 3D HCP) is replaced with better dispersed nuclei, C4′s (~5 ppm) to improve resolution and alleviate ambiguities during assignments. Multiple quantum (MQ) line narrowing effect (Grzesiek et al. 1995) is implemented to improve the sensitivity of the experiment. In the proposed experiment the intra-nucleotide peaks are efficiently suppressed whereas the inter-nucleotide peaks are enhanced. Different settings of the coherence transfer delay allow for suppression of intra-nucleotide peaks. In the cases where intra-nucleotide peaks are partially suppressed, the opposite signs of two types of peaks still make it convenient to assign them separately without any ambiguities. The experiment employs C3′/C5′ selective inversion pulses to prevent the signal modulation due to ^13^C–^13^C homonuclear couplings, these pulses also indirectly enforce the C4′/H4′ selectivity. The schematic design of the experiment is illustrated in Fig. 1 where it also compares the differences from 3D HCP experiment. Figure 1a describes the pathways for generation of both intra and inter-nucleotide peaks in 3D HCP experiment and almost unidirectional flow of magnetization due to suppression of intra-nucleotide peaks in C4′/H4′ selective 4D HC(P)CH experiment. Between the adjoining nucleotides the magnetization on ^31^P is forward transferred not only to desired C4′s, but also to other weakly coupled carbon spins, i.e. C3′ and C5′. This, collectively, causes a significant loss in sensitivity resulting in weak or undetectable resonances; such feature is not affordable in a 4D experiment. To eliminate these deleterious effects we utilized the C4′ selective inversion pulse during the coherence transfer in the experiment. Figure 1b shows a comparative illustration of the non-selective transfer in 3D HCP with the selective transfer in 4D HC(P)CH.

**Fig. 1:**
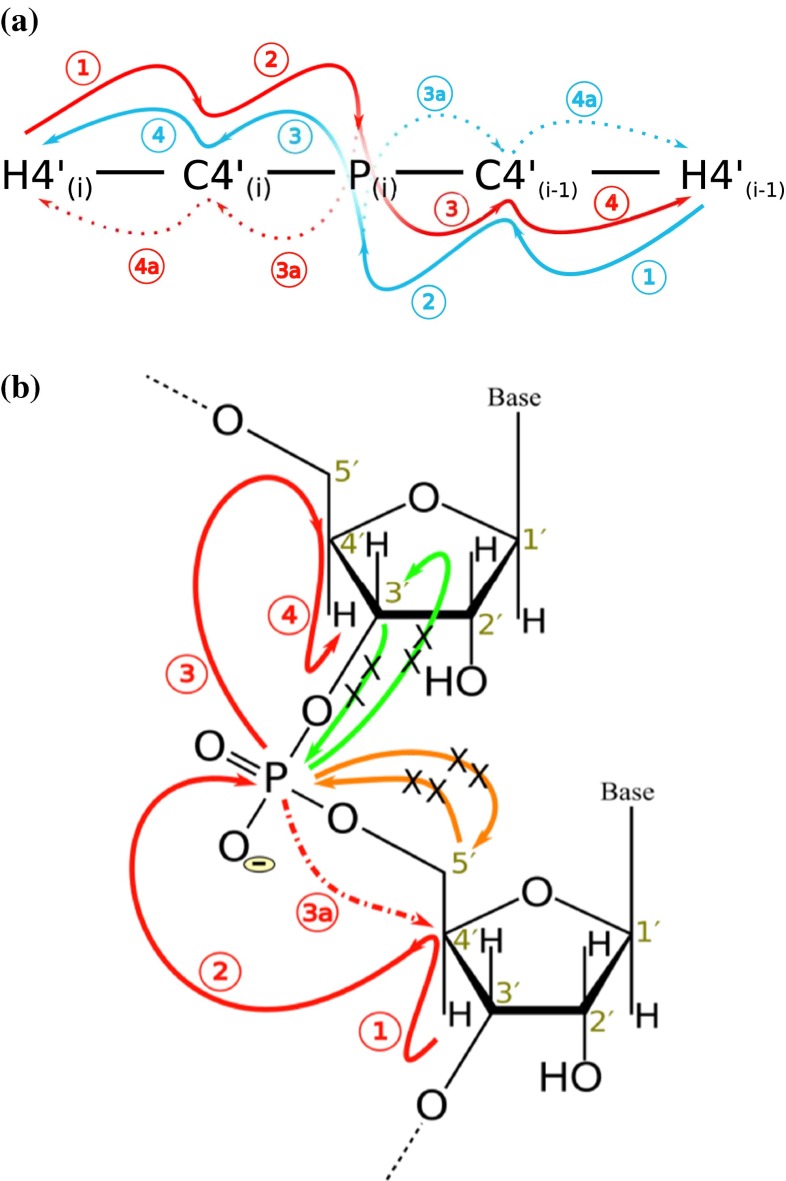
A schematic and comparative illustration of magnetization transfer in C4′/H4′ selective 4D HC(P)CH experiment. *Red* and *blue paths* represent the magnetization flow in 3′ → 5′ and 5′ → 3′ directions, respectively. The *numbers* in *circles* represent the coherence transfer steps leading to cross-peaks whereas the suffix “a” inside* circles* represents a path which generates intra-nucleotide peaks. In a 3D HCP experiment magnetization flow splits from ^31^P, generating both intra- and inter-nucleotide peaks whereas in 4D HC(P)CH experiment (**a**) intra-nucleotide peaks are suppressed (denoted by *dotted red/blue arrows*) and involving mostly unidirectional flow of magnetization. (**b**) Illustrates other key differences in coherence transfers between 3D HCP and 4D HCPCH experiments. In 3D HCP the magnetization on ^31^P gets forward transferred (P → C3′/C5′, *orange/green solid arrows*) to other sugar carbons (C3′ and C5′) whereas in 4D HC(P)CH experiment such pathways are blocked (denoted with *cross* on *orange/green arrows*); again the suppressed intra-nucleotide peak is shown by *dashed red arrow*. For the interpretation of colors in this figure the reader is referred to the online version of the Journal

The pulse scheme for C4′/H4′ selective 4D HC(P)CH experiment is shown in Fig. 2. The experiment is designed with an emphasis on achieving higher resolution with minimum sensitivity losses. High dimensionality is achieved by incorporating three indirect chemical shift evolution periods into the sequence. The pulse sequence (see Fig. 2) comprises two ^1^H–^13^C MQ periods (MQ_1_ and MQ_2_; storing MQ coherences for most of the period) and a middle ^31^P–^13^C single quantum transfer period (SQ). The magnetization flow scheme is as follows:

$$ {}_{  }^{1} {\text{H}}4 '(t_{1} )\mathop \to \limits^{{{}_{ }^{1} J _{CH }   }} {}_{  }^{13} {\text{C}}4^{ '} (t_{2} )\mathop \to \limits^{{{}_{ }^{3} J _{CP}    }} {}_{  }^{31} {\text{P}} \mathop \to \limits^{{{}_{ }^{3} J _{CP}   }}  {}_{  }^{13} {\text{C}}4 '(t_{3} )\mathop \to \limits^{{{}_{ }^{1} J _{CH }   }}  {}_{  }^{1} {\text{H}}4 '(t_{4} ) $$

**Fig. 2:**
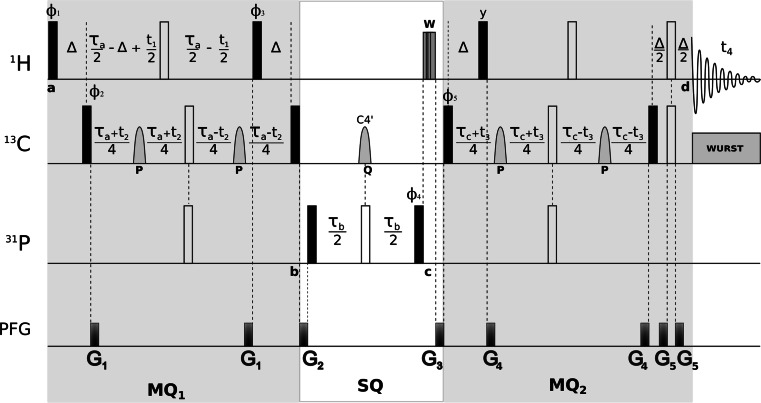
Pulse sequence scheme for through-bond, C4′/H4′ selective 4D HC(P)CH experiment. The 90° and 180° ‘hard’ pulses are represented by *filled* and *open bars*, respectively. All pulses are applied along the x-axis of the rotating frame unless indicated otherwise. *Grey sine bell-shaped* pulses (**P** and **Q**) indicate cosine modulated IBURP-2 (Geen and Freeman 1991) pulses. **P** inverts the chemical shift range 69.5 ± 6 ppm (C3′s and C5′s) with a duration of 2.5 ms (13.8 kHz peak r.f. field) and **Q** inverts the chemical shift range 83 ± 8 ppm (C4′s) with a duration of 1.9 ms (13.8 kHz peak r.f. field). **W** represent spin-lock pulses (SL_x_, SL_y_) implemented for dephasing of transverse water magnetization. ^13^C adiabatic composite pulse decoupling was performed with WURST (Kupce and Freeman 1995). The durations of ‘hard’ π/2 pulses were 7.8, 18.1 and 26.5 µs for ^1^H, ^13^C and ^31^P, respectively. Proton carrier frequency was set on resonance with water (4.68 ppm), carbon carrier was set to the centre of ^13^C4′s (83.00 ppm) and ^31^P carrier was set to −4.10 ppm. Quadrature detection in *t*_1_, *t*_2_ and *t*_3_ is accomplished by altering ϕ_1_, ϕ_2_ and ϕ_5,_ respectively, according to the States-TPPI procedure. 16-step phase cycle is as follows: ϕ_1_ = *x*; ϕ_2_ = *x*, −*x,* ϕ_3_ = 2(*y*), 2(−*y*); ϕ_4_ = 4(*x*), 4(−*x*); ϕ_5_ = 8(*x*), 8(−*x*) and ϕ_rec_ = y, 2(−*y*), *y*, 2(−*y*, 2(*y*), −*y*), *y*, (−2*y*), *y*. Delays are set as follows: ∆ = 3.5 ms ≈ (2 *J*_CH_)^−1^, τ_a_ = τ_c_ = 20.9 ms and τ_b_ = 38 ms. Gradient levels and durations are: *G*_1_ (0.2 ms, 12.7 G/cm), *G*_2_ (0.8 ms, 33.7 G/cm), *G*_3_ (1.0 ms, 42.5 G/cm), *G*_4_ (0.2 ms, 15.61 G/cm) and *G*_5_ (0.5 ms, 4.6 G/cm). A total of 1,300 (~9 %) sampling points (*t*_1_, *t*_2_, *t*_3_) were randomly chosen from a 31 × 22 × 22 Cartesian grid according to Gaussian probability distribution, *p*(*t*) = exp[−(*t/t*_max_)^2^/2σ^2^], σ = 0.5, with Poisson disk restrictions (Kazimierczuk et al. 2008). Maximum evolution times of 20 (*t*_1max_), 14 (*t*_2max_) and 14 ms (*t*_3max_) were achieved in the indirectly detected dimensions. Acquisition time was set to 85 ms (*t*_4max_). Spectral widths of 15 (ω_1_), 15 (ω_2_), 15 (ω_3_) and 12 kHz (ω_4_) were assumed. The total experiment duration was 75 h. The interscan delay of 1.8 s for optimal recovery of ^1^H magnetization (sensitivity per unit time) was used. The experiment was performed at 298 K on the Agilent DDR2 600 MHz spectrometer equipped with a room-temperature penta (^1^H/^13^C/^15^N/^2^H/^31^P) probe

Since, as it was shown earlier for nucleic acids (Fiala et al. 1998, 2000), the dominant ^1^H–^13^C dipolar relaxation mechanism is significantly attenuated for zero- and double-quantum coherences, MQ coherences are preserved during the frequency labeling of both C4′ evolution periods. In the first MQ_1_ period (see Fig. 2), the coherence starts from H4′ in sugars and is transferred to C4′ via non-refocused INEPT. H4′ and C4′ are then brought into a MQ state and the shared-evolution of chemical shifts of H4′ (*t*_1_) and C4′ (*t*_2_) is performed in a constant-time manner by shifting the corresponding hard 180° pulses within the MQ_1_ period. In order to evolve C4′–P couplings and achieve a coherence transfer, a 180° pulse on ^31^P is applied simultaneously with the C4′ inversion pulse on ^13^C channel. During this period (τ_a_), the evolution due to homonuclear carbon coupling (C4′–C3′ and C4′–C5′) is refocused by two cosine modulated IBURP-2 (Geen and Freeman 1991) pulses (**P** in Fig. 2) which selectively and simultaneously invert the frequency bands of C3′ and C5′ ribose sugar carbons. Since C2′ carbons share the same spectral region as of C3′, inversion of later also inverts the C2′ carbons. Effectively, the use of inversion pulses lead to an indirect selection of C4′ and hence H4′ during the MQ_1_ period. The next 90° pulse on H4′ and ∆ delay refocus the C4′ anti-phase to H4′ whereas the subsequent 90° pulses on C4′ and ^31^P transfer the coherence onto ^31^P.

In the next SQ period magnetization on ^31^P is brought into transverse plane and ^31^P–^13^C couplings are evolved. In the middle of this period a C4′ selective cosine modulated IBURP-2 pulse (**Q** in Fig. 2) is employed to prevent dephasing due to P_i_–C3′_i−1_ and P_i_–C5′_i_ couplings and achieve selectivity for P → C4′ transfer.

The evolution during the delay τ_b_ refocuses the P_i_–C4_i_ anti-phase and creates the P_i_–C4′_i−1_ anti-phase operators, and determines the suppression of intra-nucleotide peaks or enhancement of inter-nucleotide peaks. The suppression level of intra-nucleotide peaks is a trade-off between the J-coupling optimum delay τ_b_ and relaxation. The intensity of an intra-nucleotide peak is proportional as:

$$ {\text{I}}_{{intra({\text{C}}4^{ '}_{i} - {\text{P}}_{i} - {\text{C}}4^{ '}_{i} )}} \propto r *cos(\pi  {}_{  }\,^{3} J_{{{\text{C}}4^{ '}_{i}   {\text{P}}_{i} }}  \tau_{\text{b}} ) *cos(\pi {}_{  }\,^{3} J_{{{\text{P}}_{i} {\text{C}}4^{ '}_{i}  }}  \tau_{\text{b}} ) * e^{{ - R\tau_{\text{b}} }} $$

whereas that of inter-nucleotide peak is related as:

$$ {\text{I}}_{{inter({\text{C}}4^{ '}_{i} - {\text{P}}_{i} - {\text{C}}4^{ '}_{i - 1} )}} \propto r *sin(\pi  {}_{  }\,^{3} J_{{{\text{C}}4^{ '}_{i}   {\text{P}}_{i} }}  \tau_{\text{b}} ) *sin(\pi  {}_{  }\,^{3} J_{{{\text{P}}_{i} {\text{C}}4^{ '}_{i - 1}  }}  \tau_{\text{b}} ) * e^{{ - R\tau_{\text{b}} }} $$

where *R* is ^31^P SQ transverse relaxation rate and *r* incorporates the contributions from all other passive couplings.

The coherence transfer efficiency and hence the intensities are dependent on ^*3*^ *J*_(C4′, P)_ values (discussed later in the text). The experiments are performed at various *J* values, however at ~10 Hz we found the least loss of number of resonances in the spectrum. The transverse relaxation rate (*R*) is estimated experimentally for ^31^P and intensities for both types of peaks are plotted (see Fig. S2 in Supplementary Materials), with increasing transfer delay (τ_b_). A suitable delay (~38 ms for this study) is chosen to maximize the inter-nucleotide peak intensities, which in turn also minimizes the intra-nucleotide terms, especially in the cases where ^*3*^ *J*_(C4′i, Pi)_ ≈ ^*3*^ *J*_(Pi,C4′i−1)_.

In the consecutive block MQ_2_, the coherence is forward transferred to C4′_(i)_ where its chemical shifts are indirectly recorded (*t*_3_) preserving the MQ coherences similarly to the MQ_1_ block in the sequence. During the same period (τ_c_) refocusing of P–C4′ couplings is also achieved by application of a 180° pulse on ^31^P in synchrony with the moving 180° pulse on ^13^C channel. Another 180° pulse is centrally placed on ^1^H channel to refocus its chemical shift evolution. Again, the use of C3′/C5′ selective IBURP-2 pulses (**P** in Fig. 2) prevents the evolution due to C4′–C3′/5′ homonuclear couplings and indirectly selects C4′/H4′. Finally, an in-phase coherence is generated on H4′ spins by refocused INEPT transfer during ∆ period.

The inversion profiles for shaped pulses were simulated and tested using Spinach library (Hogben et al. 2011) on MATLAB^®^. Gradients and phase cycling are employed to eliminate undesired coherences and improve the C4′/H4′ selectivity of experiment. After P → C4′ transfer period, spin-lock pulses (SLx, SLy) are employed (**W** in Fig. 2) to dephase any remaining transverse water magnetization. The experiment complements the set of recently reported high dimensional experiments, 5D HCP-CCH COSY (Krahenbuhl et al. 2014) and 4D-NUS C_(aro)_,C_(ribo)_-NOESY (Stanek et al. 2013a), dedicated for sequential resonance assignment in RNAs.

To achieve higher dimensionality with reasonable resolution in the indirectly detected dimensions, non-uniform sampling (NUS) was employed. Using NUS we are able to acquire 4D HC(P)CH experiment with high evolution times: 20 ms (*t*_1_), 14 ms (*t*_2_) and 14 ms (*t*_3_). The processing of 4D NUS data was accomplished by the home-written software package Signal Separation Algorithm (SSA) (Stanek et al. 2012), which can be downloaded free of charge for non-commercial purposes from the website http://nmr.cent3.uw.edu.pl.

We have tested the performance of C4′/H4′ selective 4D HC(P)CH experiment on a fairly demanding RNA sample which encompasses typical structural elements. The experiments were run on a ^13^C,^15^N-labeled 34-nt hairpin RNA (1.5 mM in D_2_O solution) consisting of two A-RNA form stems, one adenine bulge, an asymmetric internal loop and a GAAA terminal loop (Cevec et al. 2010). The 4D HC(P)CH spectrum was easily analyzed with SPARKY (Goddard and Kneller 2004) program by synchronizing two dimensions (H4′ and C4′) of *i*th nucleotide (see Figs. 3, S3 in Supplementary Materials); the resulting 2D plane consists of inter-nucleotide peaks, i.e. to the (*i*−1)th and (*i*+1)th nucleotides. In other words, to achieve the sequential assignment, H4′C4′ plane of one nucleotide is correlated with the H4′C4′ planes of two neighboring nucleotides. Figures 3, S3 show the representative 2D planes of 4D HC(P)CH spectrum illustrating the resolution advantage in the experiment. It can clearly be seen that heavily overlapped peaks (e.g. C33, U32, C31, A28, C29, A20, G19) in 2D ^13^C-HSQC (Fig. 3a) are clearly resolved in 4D experiment along the H4′C4′ planes of adjoining nucleotides (Figs. 3b–d, S3a–d). To compare the suppression levels of intra-nucleotide peaks, another 4D experiment was acquired without emphasis on suppression, i.e. using τ_b_ ~22 ms. The 2D planes from this version of experiment consist of one intra- and two inter-nucleotide peaks (of course, with an exception for terminal nucleotide). As can be expected, in this version of experiment intra-nucleotide peaks are more intense than inter-nucleotide peaks (see Fig. 3e–g). A significant suppression of intra- and enhancement of inter-nucleotide peaks can be compared between the two versions of experiment in Fig. 3 (b, e), (c, f) and (d, g) pairs. In the cases of incomplete suppression of intra-nucleotide peaks, their opposite sign still reduces ambiguities during assignments.

**Fig. 3:**
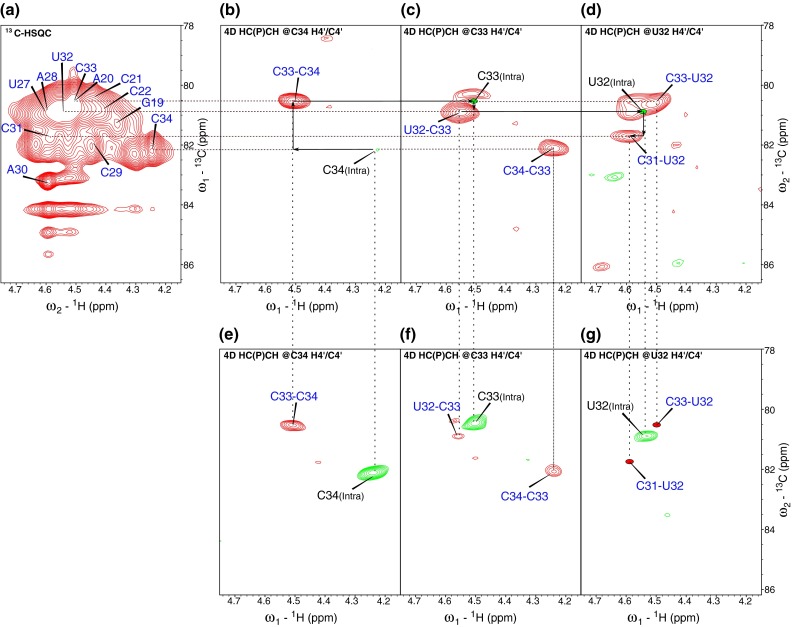
Representative cross-sections from 4D HC(P)CH experiment. **(a)** shows the overlapped H4′C4′ region of 2D ^13^C-HSQC spectrum. Resolution enhancement can be seen in (**b**–**d**) which are the 2D cross-sections of 4D HC(P)CH spectrum extracted along the H4′C4′ dimensions of C34, C33, U32 respectively. The peaks are clearly resolved in the H4′C4′ plane, enabling an unambiguous assignment of cross-peaks to the neighboring nucleotides. For example, the assignment of C34–C33, C33–U32, U32–C31 inter-nucleotide peaks (marked in *blue*) is achieved based on the H4′C4′ planes of C34 (**b**), C33 (**c**), U32 (**d**) respectively. Intra-nucleotide-peaks are labeled in *grey*. Also illustrated is the comparison between 4D HC(P)CH experiments with (**b**–**d**) and without (**e**–**g**) suppression of intra-nucleotide peaks. For the non-suppressed version of experiment each 2D cross section (**e**–**g**) contains one intra-nucleotide peak (*green contours*) and two inter-nucleotide peaks (*red contours*), i.e. to the previous and the next nucleotide, respectively. *Dotted vertical lines* in the (**b**, **e**), (**c**, **f**) and (**d**, **g**) pairs compare the suppression of intra-nucleotide peaks and enhancement of inter-nucleotide peaks between two versions of the experiment. Since C34 is the terminal nucleotide, only one inter-nucleotide peak is observed in its C/H plane (**b**, **e**). The position of completely suppressed intra-nucleotide peaks is indicated by *solid green dots* (**c**, **d**) whereas inter-nucleotide peaks below detection limit are indicated by *solid red dots* (**g**). For the interpretation of colors in this figure the reader is referred to the online version of the Journal

Overall, 19 sequential connectivities were successfully established (see Fig. 4) using C4′/H4′ selective 4D HC(P)CH whereas 3D HCP experiment could provide only 4 sequential links in 34-nt RNA. Comparatively, the previously reported 4D C_(aro)_,C_(ribo)_-NOESY experiment provided 17 sequential links, which reflects the difficulty of the investigated RNA sample. Interestingly, 4D HC(P)CH and 4D NOESY experiments provided complementary data for sequential assignment. In combination, 26 (out of 33) sequential links were successfully assigned. The missing assignments are either due to structural mobility, manifesting in enhanced relaxation during coherence transfer periods or due to small C4′–P couplings. In our previous study we have shown that, in this RNA, the asymmetric internal loop adopts two energetically comparable families of structures, which both satisfy NMR data (Cevec et al. 2010). In addition, the C4′ → P/P → C4′ transfers in 4D HC(P)CH experiment rely on the C4′–P couplings (^3^ *J*_C4′,P_), which in turn depend on the β/ε torsional angles in RNA (Schwalbe et al. 1994; Legault et al. 1995; Hu et al. 1999). For RNA used in this study (PDB ID: 2KPV) the coupling constants (^3^ *J*_C4′,P_) were calculated based on the parameterized Karplus equation (Mooren et al. 1994). It can be observed that for some of the cases the C4′–P couplings are very small (see Fig. 5) and an efficient coherence transfer is difficult to achieve. It is noteworthy that, in this study, most of the missing resonances relate to the internal loop or to the proximate residues where the β/ε angles are large and therefore C4′–P couplings are very small.

**Fig. 4:**
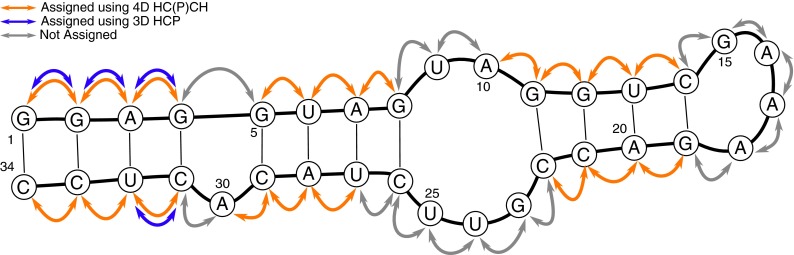
The schematic presentation of the investigated 34-nt RNA showing the sequential connectivities observed in the 3D HCP and 4D HC(P)CH spectrum. *Blue arrows* indicate the sequential links assigned using 3D HCP experiment, while *orange arrows* indicate sequential connectivities obtained from C4′/H4′ selective 4D HC(P)CH experiment. Very weak or missing correlations are marked with the *grey arrows*, most of which belong to internal loop or to the proximate residues

**Fig. 5:**
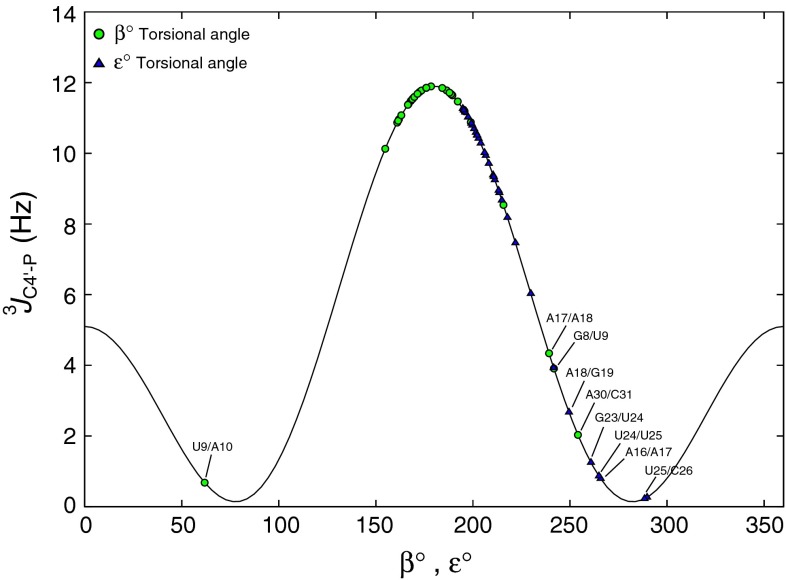
Coupling constant versus β and ε torsional angles of 34-nt RNA (PDB ID: 2KPV). *Thin solid line* is the Karplus curve of ^*3*^ *J*_C4′–P_ based on the parameterized Karplus equation (Mooren et al. 1994). The coupling constant values based on β angles (5′ → 3′, ^3^ *J*_C4′–P_) are indicated by *green circles* while those obtained from ε angles (3′ → 5′, ^3^ *J*_C4′–P_) are indicated by *blue triangles*. The unfavorable or week couplings (large β/ε angles), as labeled, mostly belong to internal loop or to the proximate residues

To conclude, we have introduced a through-bond, C4′/H4′ selective, non-uniformly sampled 4D HC(P)CH experiment for sequential assignments in RNAs. The incorporated indirect dimensions, along with the replacement of evolution of ^31^P by ^13^C4′, significantly enhanced the spectral dispersion. NUS was employed to achieve high resolution in all the indirectly detected dimensions. Band selective inversion pulses were used to prevent signal modulation due to C4′–C3′, C4′–C5′ couplings and to indirectly select the C4′/H4′ region. Experiment involved the suppression of intra-nucleotide peaks, as a result, the number of ambiguities were further reduced. We have demonstrated that the C4′/H4′ selectivity and attenuated relaxation of MQ coherences partially compensated the sensitivity losses entailing the increased dimensionality. Despite lower sensitivity, the proposed experiment clearly outperforms the conventional HCP experiment, which suffers from critical overlap in the “linking” ^31^P dimension. The experiment is proposed as a complementary tool to 3D/4D NOESY experiments and augments the set of high dimensional experiments aimed at improving resolution and reducing ambiguities during resonance assignments in RNAs with poor chemical shift dispersion.

## Electronic supplementary material

Below is the link to the electronic supplementary material.

Supplementary material 1 (PDF 751 kb)
