# Supplementary material for: C4′/H4′ selective, non-uniformly sampled 4D HC(P)CH experiment for sequential assignments of 13C-labeled RNAs
Source: J Biomol NMR. 2014 Sep 10;60(2):91–8. doi: 10.1007/s10858-014-9861-z (PMC4207962; doi:10.1007/s10858-014-9861-z)
Supplement: Supplementary file 1 — Supplementary material 1 (PDF 751 kb) [file 10858_2014_9861_MOESM1_ESM.pdf]

C4'/H4' selective, non-uniformly sampled 4D HC(P)CH experiment for sequential assignments of ^{13}C-labeled RNAs

Saurabh Saxena[1], Jan Stanek[1], Mirko Cevec[2], Janez Plavec[2,3,4], Wiktor Koźmiński[1,*]

[1]*Biological and Chemical Research Centre (CENT III), Faculty of Chemistry, University of Warsaw, Pasteura1, 02093, Warsaw, Poland*

[2] *Slovenian NMR Centre, National Institute of Chemistry, Hajdrihova ulica 19, 1000 Ljubljana, Slovenia*

[3] *EN-FIST Centre of Excellence, Dunajska cesta 156, 1000 Ljubljana, Slovenia*

[4] *Faculty of Chemistry and Chemical Technology, University of Ljubljana, Aškerčeva cesta 5, 1000 Ljubljana, Slovenia*

Supporting Information

* to whom the correspondence should be addressed

kozmin@chem.uw.edu.pl

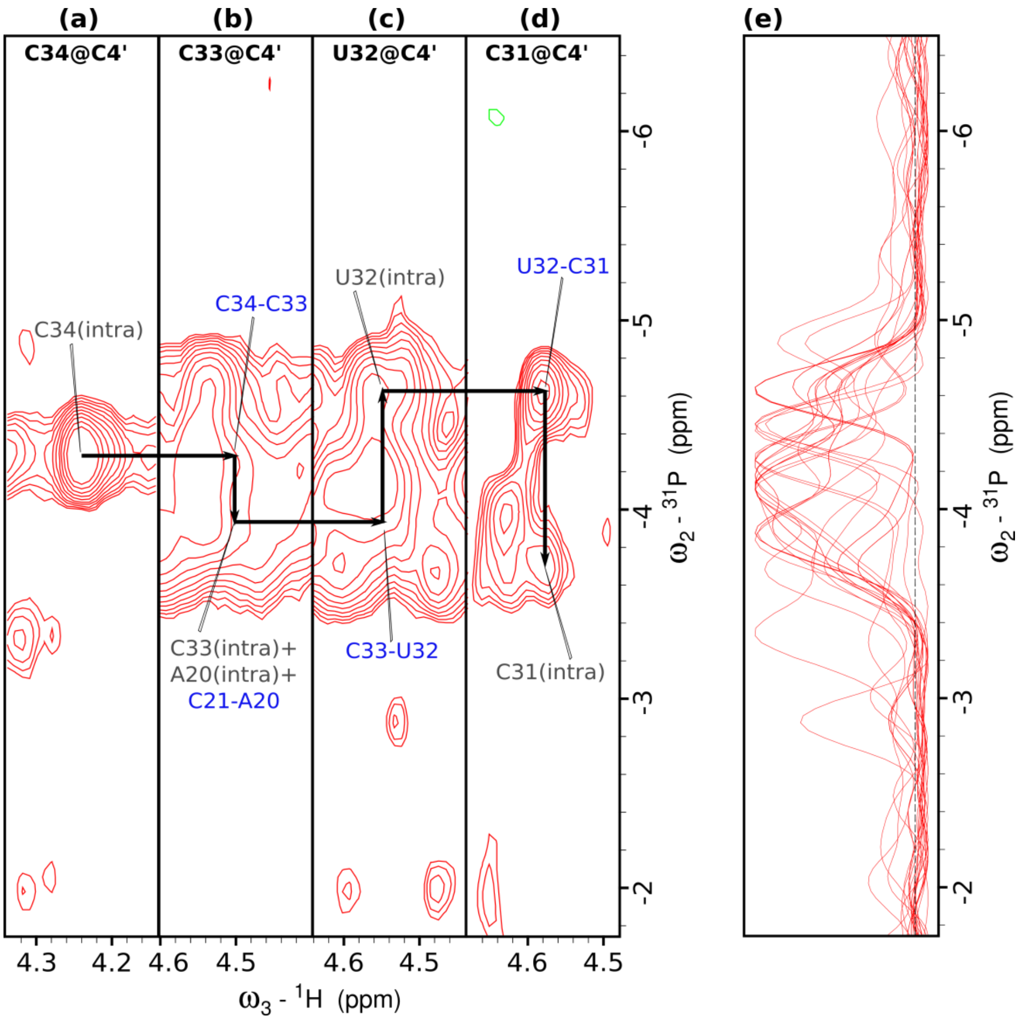

Fig. S1 Spectral overlap, ambiguities and low ^{31}P chemical shift dispersion in 3D HCP experiment. (**a**) to (**d**) are strip plots of 3D HCP spectrum extracted along C4' of corresponding nucleotide. Arrows show the pathway for achieving the sequential resonance assignment. (**b**) and (**c**) show severe overlaps between inter-nucleotide peaks (marked in blue) exposed to additional crowding due to intra-nucleotide peaks (marked in grey). (**e**) shows collective 1D traces across ^{31}P dimension illustrating its poor chemical shift dispersion (~1.8 ppm).

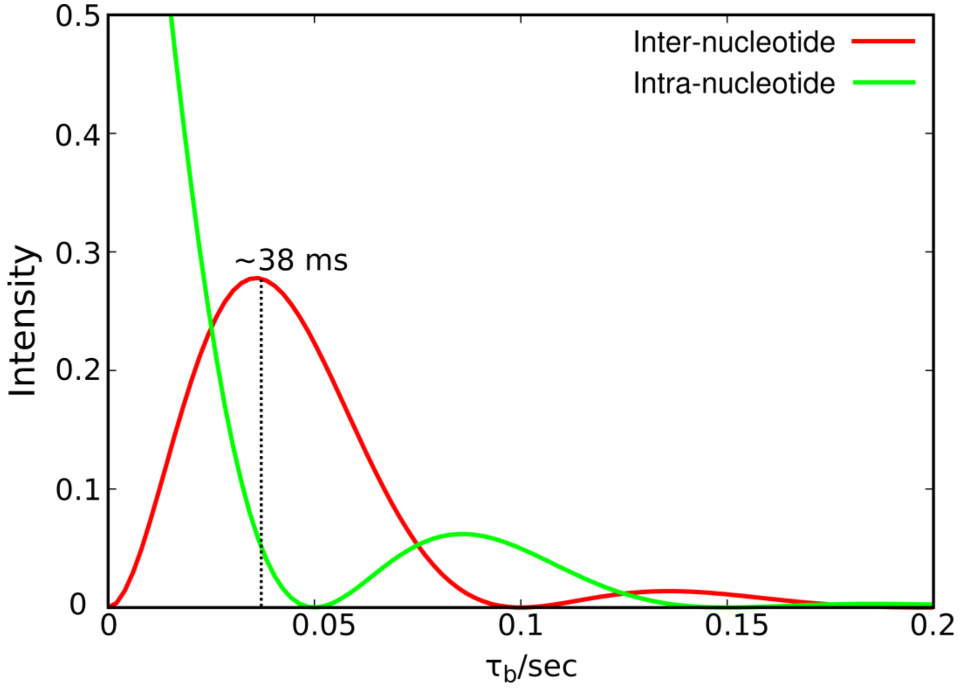

Fig. S2 Inter- and intra-nucleotide peak intensities as a function of P-C4' transfer delay (τ_b). The intensities were calculated with experimentally estimated ^{31}P transverse relaxation (~30 Hz) and P-C4' couplings ($^3J_{P\text{-}C4'}$, ~10 Hz). A suitable delay (~38 ms) is chosen to maximize the inter-nucleotide peak intensities (red curve) and minimize intra-nucleotide peak intensities (green curve).

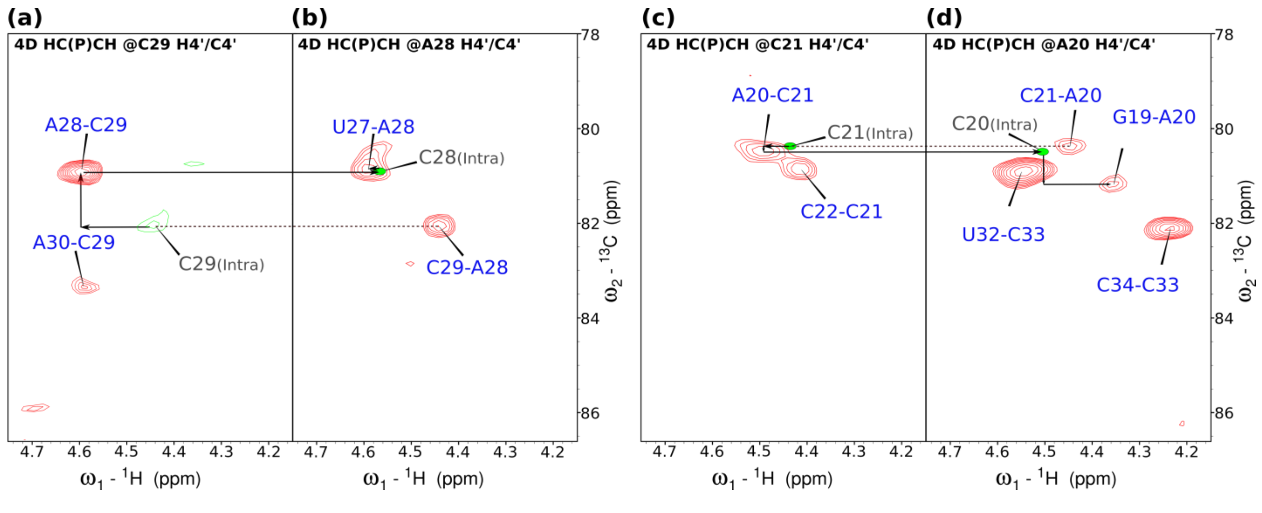

Fig. S3 Representative 2D ω_1-ω_2 cross-sections from 4D HC(P)CH spectrum. Resolution enhancement can be seen in (**a**)-(**d**) planes which are extracted along the H4'/C4' dimensions of C29, A28, C21 and A20, respectively**.** The peaks are clearly resolved in the C4'H4' plane, enabling an unambiguous assignment of cross-peaks to the neighboring nucleotides. For example, the assignment of A30-C29, C29-A28, A28-U27, C22-C21, C21-A20 and A20-G19 inter-nucleotide peaks (marked in blue) is achieved based on the H4'C4' planes of C29(**a**), A28(**b**), C21(**c**) and A20(**d**), respectively. Intra-nucleotide-peaks are labelled in grey. For illustration, the positions of completely suppressed intra-nucleotide peak are indicated with solid green dots. For the interpretation of colors in this figure the reader is referred to the online version of the Journal.
